# Chylothorax following endovascular aortic repair with subclavian revascularization - a case report

**DOI:** 10.1186/s13019-014-0165-x

**Published:** 2014-11-01

**Authors:** Yuan-Jang Hsu, Pin-Ru Chen, Yu-Sen Lin, Hsin-Yuan Fang, Chien-Kuang Chen

**Affiliations:** Division of Thoracic Surgery, Department of Surgery, China Medical University Hospital, Taichung, Taiwan; Graduate Institute of Clinical Medical Science, China Medical University, Taichung, Taiwan; School of Medicine, China Medical University, Taichung, Taiwan; China Medical University, 2 Yuh-Der Road, Taichung, 404 Taiwan

## Abstract

**Background:**

Thoracic endovascular aortic repair (TEVAR) is becoming increasingly popular due to reduced perioperative morbidity and mortality compared with open surgical repair. However, complications can occur when the left subclavian artery is involved. When performing TEVAR with left carotid-subclavian artery bypass the stent graft will extend to the left common carotid artery. We herein present the case of a patient with a type B aortic dissection with an acute intramural hematoma. Chylothorax was noted after TEVAR with left carotid-subclavian artery bypass.

**Case report:**

A 66-year-old female with descending aortic dissection that was treated conservatively developed the sudden onset of back pain. Aortic computed tomography (CT) showed a type B intramural aortic dissection. TEVAR with left carotid-subclavian artery bypass was performed. Left chylothorax was noted after surgery with drainage of up to 1000 mL per day. Conservative management was ineffective. Thoracoscopic ligation of the thoracic duct was performed with resolution of the chyle leakage.

**Conclusion:**

Chylothorax can occur after TEVAR with carotid-subclavian artery bypass and likely results from thoracic duct injury. When conservative treatments fail, ligation of the thoracic duct cephalad to aortic hiatus can resolve the chyle leakage.

**Electronic supplementary material:**

The online version of this article (doi:10.1186/s13019-014-0165-x) contains supplementary material, which is available to authorized users.

## Background

Thoracic endovascular aortic repair (TEVAR) is becoming increasingly popular due to reduced perioperative morbidity and mortality compared with open surgical repair [1]. We herein present the case of a patient with a type B aortic dissection with an acute intramural hematoma. Left chylothorax was noted after TEVAR with left carotid-subclavian artery bypass.

## Case presentation

**Figure 1:**
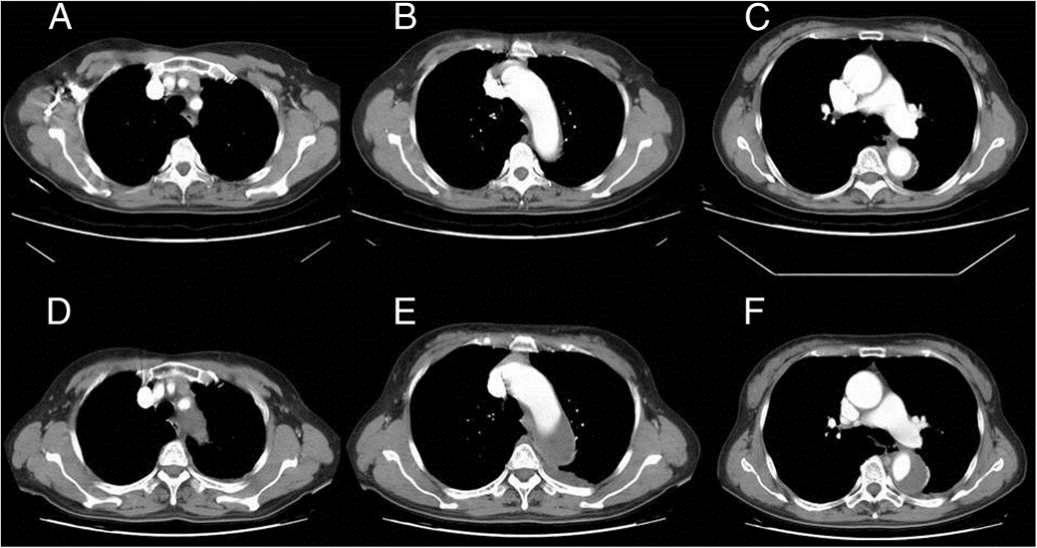
**Transverse CT scan showed hematoma formation in the descending aorta. (A-C)** One month before admission, descending aortic dissection was diagnosed and the patient was treated with medical therapy. **(D-F)** At admission, as compared to the prior CT scan an intramural hematoma with false lumen was noted.

**Figure 2:**
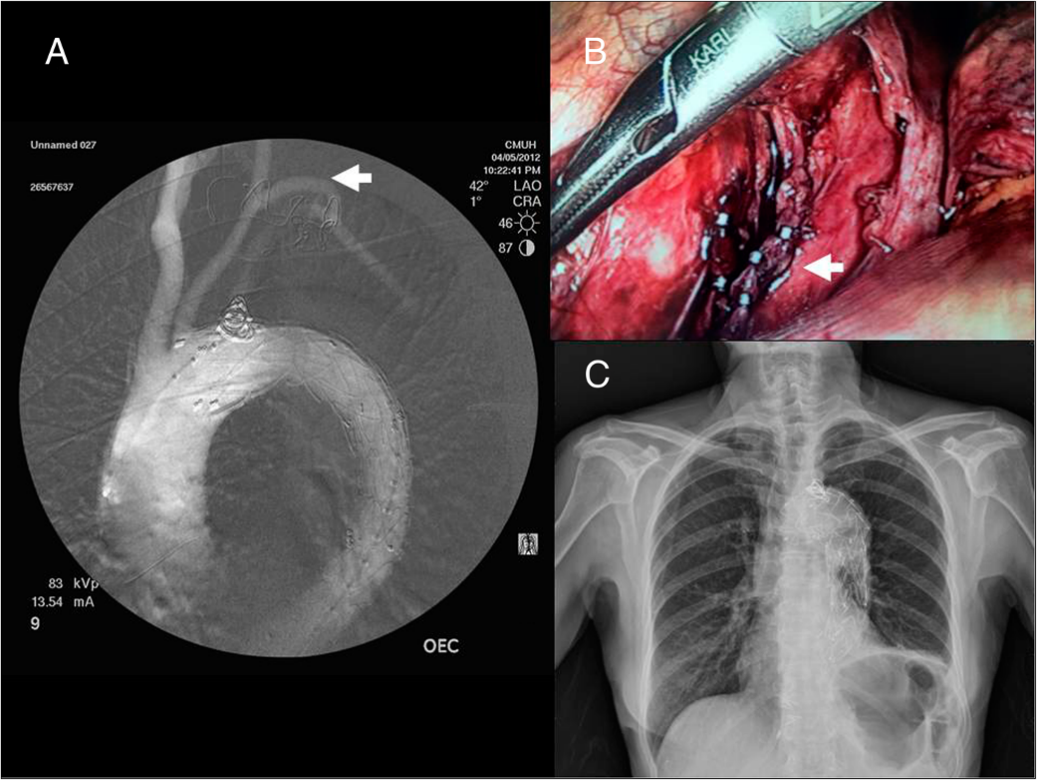
**Images in operation and following. (A)** The left subclavian artery was covered, and the stent graft extended to the left common carotid artery. A coil was placed in the proximal left subclavian artery. A right to left carotid-carotid bypass was performed (arrow). **(B)** Thoracoscopic surgery was performed to dissect and ligate the thoracic duct (arrow). **(C)** No pleural effusion was noted at the 3-month follow-up.

A 66-year-old female developed a descending aortic dissection and was treated conservatively with medical therapy because there were no complications such as splanchnic ischemia, renal insufficiency, lower extremity ischemia, or focal neurologic deficits. One month later she was admitted via our emergency department (ED) because of recurrent severe back pain. Aortic computed tomography (CT) showed a type B aortic dissection with intramural hematoma and false lumen expansion (Figure 1). She underwent TEVAR. Left carotid-subclavian artery bypass was performed by exploration from left low neck for left subclavian artery coverage (Figure 2A). After surgery, she resumed oral intake and increased left pleural drainage of a milky fluid was noted on postoperative day 2. Aortic CT on postoperative day 4 revealed no endoleak or thrombosis. Persistent pleural drainage of a milky appearing fluid of more than 1000 mL per day continued. The pleural fluid triglyceride concentration was 227 mg/dL. Left chylothorax resulting from disruption of the thoracic duct during the left carotid-subclavian artery bypass was suspected. Conservative medical treatment of nothing per oral (NPO) and administration of total parenteral nutrition (TPN) failed to reduce the amount of drainage. Therefore, surgical ligation of the thoracic duct was planned at postoperative day 30. After induction of general anesthesia, the right lung was deflated, and 3 trocar sleeves were inserted at lateral ninth, anterior lateral seventh, and anterior lateral fifth intercostal spaces. The pulmonary ligament was transected, and the lung was retracted. The parietal pleura was incised along the distal part of azygos vein toward diaphragm. The thoracic duct should be found between aorta and azygos vein. Careful dissection exposed the thoracic duct as near as possible to diaphragm, which was then ligated (Figure 2B). Her recovery was uneventful, and the left Jackson-Pratt drain was removed on postoperative day 5. At the 3-month follow-up, chylothorax was not recurrent (Figure 2C).

## Discussion

TEVAR was developed to address degenerative aneurysmal disease including aortic dissection, aortic transection, intramural hematoma, and penetrating aortic ulcer [1],[2]. When treating proximal lesions, the left subclavian artery will be covered and the stent graft will extend to the left common carotid artery [3],[4]. The principal branches arising from the subclavian artery are the thyrocervical trunk, which gives rise to the ascending and transverse cervical arteries, the suprascapular artery, and the inferior thyroid artery; the costocervical trunk; the internal mammary artery; and the vertebral artery. Thus, there are many potential complications following subclavian artery occlusion including upper extremity ischemia, stroke, spinal cord ischemia, and myocardial ischemia [3]-[7]. Carotid-subclavian artery bypass was designed to resolve subclavian artery occlusion. Unfortunately, many complications following carotid-subclavian artery bypass can occur including vocal cord paralysis, phrenic nerve palsy, vagus nerve injury, bleeding, thoracic duct injury, lymphocele, and sympathetic nerve injury resulting in Horner syndrome [7]-[10].

Chylothorax results from disruption or obstruction of the thoracic duct and subsequent leakage of chyle (lymphatic fluid of intestinal origin) into the pleural space [11]. The pleural effusion typically has a high triglyceride concentration, and often a turbid or milky white appearance. The etiologies of chylothorax can be categorized as nontraumatic or traumatic [12],[13]. Malignancy is the leading cause of nontraumatic chylothorax. Surgical procedures in the area of the thoracic duct or nearby structures can result in disruption of the thoracic duct or shearing of lymphatic tributaries, and account for the majority of traumatic chylothorax cases.

The thoracic duct is the largest lymph vessel, and extends from the second lumbar vertebra to the root of the neck. It enters the thorax through the aortic opening of the diaphragm between the aorta and the azygos vein. At the level of the fifth thoracic vertebra, the thoracic duct inclines towards the left side to enter the superior mediastinum and ascends behind the aortic arch and the thoracic part of the left subclavian artery, between the left side of the esophagus and the left pleura, to the thoracic inlet. In the neck, the arch of the thoracic duct rises 3 or 4 cm (up to 6 cm) above the clavicle and curves anterior to the vertebral artery and vein, the left sympathetic trunk, the thyrocervical artery or its branches, the left phrenic nerve, and the medial border of scalenus anterior. It then passes posterior to the left common carotid artery, vagus nerve, and internal jugular vein, and finally ends by opening into the angle of the junction of the left subclavian vein and the internal jugular vein.

Shimado and Sato reported four types of thoracic duct terminations [14]. Type A (38%) terminated at the venous angle, type B (27%) at the terminal end of the internal jugular vein, type C (28%) at the terminal end of the external jugular vein, and type D (7%) was described as a complex branching pattern. Each type was further subdivided depending upon the number of terminal branches, some of which drained directly into the subclavian vein. The termination is usually (87.5 to 100% of the time) within 1 cm of the venous angle [15]. Langford and Daudia reported 21 ducts (87.5%) terminated as a single vessel, two ducts (8.33%) showed a bifid termination, and one duct (4.2%) had three terminal branches. Five thoracic ducts (20.8%) showed branching and re-anastamosing patterns prior to their termination, irrespective of the number of terminal branches [16]. The presence of such complex patterns is an additional risk factor for iatrogenic trauma, and a potent source of confusion when faced with the surgical management of a chyle leak.

When TEVAR is performed at the proximal portion of the aorta, as in our patient, the left subclavian artery will be covered by the membrane portion of the stent graft and the subclavian artery will be occluded. Left carotid-subclavian artery bypass is performed at the same time. The thoracic duct drains chyle into the blood stream via the left subclavian vein. It is possible to injury the thoracic duct when performing the bypass, to result in chyle leakage. We believe this is what occurred in our patient.

For patients with traumatic chylothorax, initial conservative management rather than surgical intervention is recommended. Conservative management includes chest tube drainage, and either bowel rest with total parenteral nutrition (TPN) or a high protein-reduced fat diet with medium chain triglyceride (MCT) supplementation [17],[18]. Attention to fluid and electrolyte management, nutrition, and the daily volume of pleural drainage are necessary. If the amount of chylous fluid drainage is >1 L per day through the chest tube, early thoracic duct ligation is recommended. Patients draining more than 1 L/day are unlikely to respond to conservative therapy, and usually require surgical intervention within 5 to 7 days [19]. In contrast, patients with?<?500 mL of chest tube drainage in the first 24 hours after cessation of oral intake and initiation of TPN tend to improve with conservative management [20]. If pleural drainage continues after 14 days of conservative therapy, some studies recommand thoracic duct ligation as a longer duration of conservative therapy is associated with nutritional depletion and high mortality rates [21],[22].

The advent of thoracoscopic surgery over the last decade has changed the approach to the management of a number of chest diseases. Because of fewer and smaller incisions, thoracoscopic surgery is associated with less pain, faster postoperative recovery, shortened hospital stay, and decreased long-term morbidity. Intrathoracic postoperative adhesions are milder with thoracoscopic surgery compared to open thoracotomy. Thoracoscopic surgery has the advantage of shortened recuperation time and decreased blood loss [23]-[25]. Because of the benefits associated with thoracoscopic surgery, we utilized a thoracoscopic approach and the thoracic duct was reached through the right pleural cavity, and ligated just cephalad to the aortic hiatus. Postoperatively, there was no chylothorax recurrence in our patient during the 3 months follow-up.

## Conclusion

Subclavian revascularization procedures can be performed with relatively low risk, and complications are rare. Chylothorax can occur after TEVAR with carotid-subclavian artery bypass from neck exploration and likely results from thoracic duct injury. When conservative treatments fail, ligation of the thoracic duct cephalad to aortic hiatus by thoracoscopic surgery can resolve the chyle leakage.

## Consent

Written informed consent was obtained from the patient for publication of this case report and accompanying images. A copy of the written consent is available for review by the Editor-in-Chief of this journal.

## Authors' contributions

YJH carried out the manuscript. PRC collected references. YSL took the pictures of the case report. HYF performed the operation of the patient. CKC coordinated all authors and revised the manuscript. All authors read and approved the final manuscript.

## Authors’ original submitted files for images

Below are the links to the authors’ original submitted files for images. Authors’ original file for figure 1 Authors’ original file for figure 2
