# Supplementary figures and images for: Chylothorax following endovascular aortic repair with subclavian revascularization - a case report
Source: J Cardiothorac Surg. 2014 Nov 1;9:165. doi: 10.1186/s13019-014-0165-x (PMC4234843; doi:10.1186/s13019-014-0165-x)

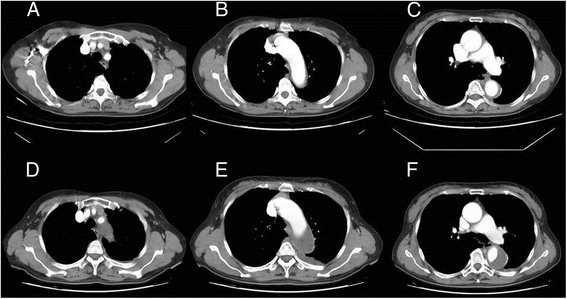

Supplement: Supplementary file 1 — Authors’ original file for figure 1 [file 13019_2014_165_MOESM1_ESM.gif]

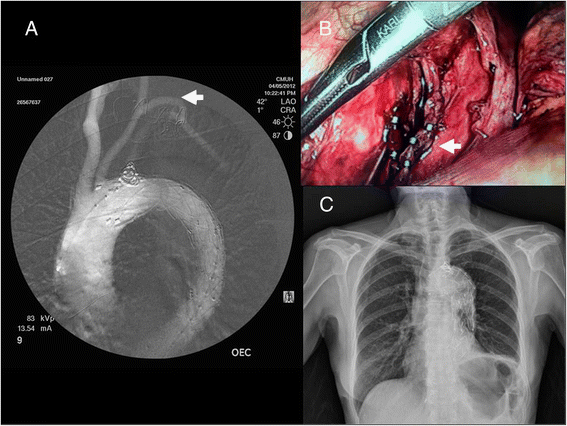

Supplement: Supplementary file 2 — Authors’ original file for figure 2 [file 13019_2014_165_MOESM2_ESM.gif]
